# Technical and dosimetric considerations in IMRT treatment planning for large target volumes

**DOI:** 10.1120/jacmp.v6i4.2129

**Published:** 2005-11-22

**Authors:** Harish K. Malhotra, Sanjay Raina, Jaiteerth S. Avadhani, Steven deBoer, Matthew B. Podgorsak

**Affiliations:** ^1^ Department of Radiation Medicine Roswell Park Cancer Institute Buffalo New York 14263 U.S.A.

**Keywords:** large‐field IMRT, treatment planning, step and shoot IMRT, sliding window IMRT

## Abstract

The maximum width of an intensity‐modulated radiotherapy (IMRT) treatment field is usually smaller than the conventional maximum collimator opening because of design limitations inherent in some multileaf collimators (MLCs). To increase the effective field width, IMRT fluences can be split and delivered with multiple carriage positions. However, not all treatment‐planning systems and MLCs support this technique, and if they do, the maximum field width in multiple carriage position delivery is still significantly less than the maximum collimator opening. For target volumes with dimensions exceeding the field size limit for multiple carriage position delivery, such as liver tumors or other malignancies in the abdominal cavity, IMRT treatment can be accomplished with multiple isocenters or with an extended treatment distance. To study dosimetric statistics of large field IMRT planning, an elliptical volume was chosen as a target within a cubic phantom centered at a depth of 7.5 cm. Multiple three‐field plans (one AP and two oblique beams with 160° between them to avoid parallel opposed geometry) with constraints designed to give 100% dose to the elliptical target were developed. Plans were designed with a single anterior field with dual carriage positions, or with the anterior field split into two fields with separate isocenters 8 cm apart with the beams being forcibly matched at the isocenter or with a 1 cm, 2 cm, 3 cm, and 4 cm overlap. The oblique beams were planned with a single carriage position in all cases. All beams had a nominal energy of 6 MV. In the dual isocenter plans, jaws were manually fixed and dose constraints remained unaltered. Dosimetric statistics were studied for plans developed for treatment delivery using both dynamic leaf motion (sliding window) and multiple static segments (step and shoot) with the number of segments varying from 5 to 30. All plans were analyzed based on the dose homogeneity in the isocenter plane, 2 cm anterior and 2 cm posterior to it, along with their corresponding dose‐volume histograms (DVHs). All the dual isocenter plans had slight underdosage anterior to the match point and slight overdosage posterior to it, while the dual carriage plan had a nice blending of the dose distribution without the accompanying hot or cold spots. Based on the dose statistics, it was noted that the dual isocenter plans can be clinically acceptable if they have at least a 3‐cm overlap. In the case of step and shoot IMRT, the number of segments used in a dual carriage plan was found to affect the overall plan dosimetric indices.

PACS number: 87.53.Tf

## I. INTRODUCTION

Intensity‐modulated radiotherapy (IMRT) is rapidly becoming the treatment of choice for delivery of highly conformal radiation therapy. Both static (also known as step and shoot) and dynamic (sliding window) methods of IMRT dose delivery have been developed.^(^ ^1^ ^–^ ^3^ ^)^ In IMRT treatment planning, the necessary beam orientations are specified, and dose constraints are defined for all relevant structures. The planning system then determines an optimal fluence for each field that best satisfies the defined constraints. This optimal fluence is subsequently converted into a deliverable fluence taking into account the physical limitations of the multileaf collimator (MLC) that is to be used in treatment delivery.

In evaluating field size limitations in IMRT, it is necessary to understand some important MLC design features in addition to how IMRT dose delivery proceeds. First, the separation between the fully extended and fully retracted leaves on any one bank, known as the leaf span, is limited by the physical length of each leaf. Second, there may be a limitation on how far an individual leaf can travel over the beam central axis. Finally, during IMRT, the main collimator is usually positioned to shield abutting leaf ends to avoid excessive leaf‐end transmission. For the MLC (52‐leaf, Mark Series, Varian Associates, Palo Alto, CA) used in our facility, the leaf span is 14.5 cm, the maximum leaf travel over the beam central axis is 16 cm, and the abutting leaf junction must be 5 mm from the projected Xl‐jaw edge, all measured within the isocenter plane. During IMRT treatment delivery, MLC leaves initially define subfields on one side of the target volume (toward the X1‐jaw) and move toward the opposite side (X2‐jaw). This scheme is used irrespective of whether the treatment is delivered using the step and shoot or the sliding window approach. Taking into account the limitations described above, Fig. 1(a) shows that the maximum width that can be treated by a single IMRT field is 14.0 cm, with the requirement that the treatment isocenter be within the irradiated volume. To treat larger target volumes, advantage is taken of the fact that each MLC bank is mounted on a carriage. Both carriages can move in the same plane as the X‐jaws and could, in principle, be moved to sequentially deliver fluences that result in IMRT fields wider than 14.0 cm. As shown in Figs. 1(b) to (d), however, even with dual and triple carriage position treatment delivery, IMRT field widths are limited to 26 cm (or 25.5 cm, depending on treatment location relative to isocenter) and 31.5 cm, respectively.

**Figure 1(a):**
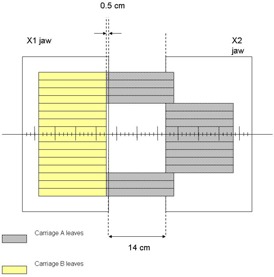
Schematic diagram showing the maximum IMRT treatment field width achievable with a single carriage position using the MLC installed at our facility. The collimator jaws that move in the same plane as the MLC are also depicted. For clarity of presentation, only a subset of the entire leaf bank of 26 leaves on both carriages is displayed.

**Figure 1(b):**
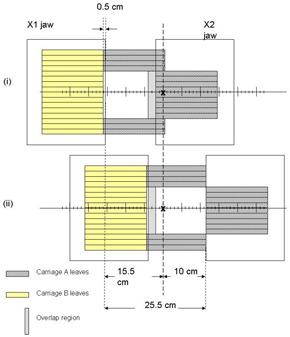
Schematic diagram showing the maximum IMRT treatment field width achievable with dual carriage position delivery. The treatment isocenter is placed such that leaves outside the target region are extended 16 cm across the central axis plane. The first and second carriage positions are depicted as (i) and (ii), with an overlap region as drawn.

**Figure 1(c):**
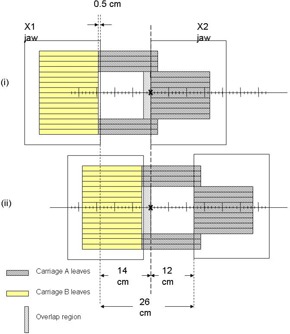
Schematic diagram showing the maximum IMRT treatment field width achievable with dual carriage position delivery. In this geometry, the treatment isocenter is placed so that leaves outside the target region do not extend the full 16 cm across the central axis plane. The two carriage positions are depicted in (i) and (ii) with their overlap region as shown.

**Figure 1(d):**
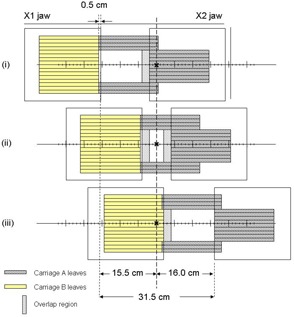
(d). Schematic diagram showing the maximum IMRT treatment field width achievable with triple carriage position delivery. The three carriage positions are depicted in (i), (ii), and (iii), with respective overlaps as shown.

The method of subdividing a large fluence into multiple subfields with different carriage positions was originally proposed by Wu et al.^(4)^ and uses a feature called “dynamic feathering,” where the component beams overlap by a small amount. In the overlap region, which is usually 2 cm, the intensity of one subfield continuously decreases while that of the other adjacent subfield increases. This can be easily achieved in the case of a dynamic IMRT treatment, which usually has a few hundred control points (or subfields) per field. In step and shoot IMRT dose delivery, the number of segments per field is much less and in many cases approaches one‐tenth the number used in dynamic IMRT dose delivery. Thus, theoretically, the homogeneity in the overlap region will depend on the particular treatment delivery technique and the number of subfields used.

To treat field widths larger than 31.5 cm with an IMRT technique, it is necessary to either extend the treatment distance (source‐to‐surface distance, SSD) or to use multiple‐field isocenters. The clinical motivation for the study comes from a patient with liver carcinoma who we treated recently with IMRT. The treatment volume was larger than what was possible to treat even with triple carriage IMRT setup. Furthermore, we are aware of at least one commercially available IMRT treatment planning system that does not have an algorithm to split a large IMRT field into dual and triple carriage positions and necessitates the use of multiple isocenters even for moderate‐size tumors. A dosimetric analysis of the use of multiple isocenters for an IMRT treatment is presented below. The issue of IMRT treatment at extended SSD is discussed afterward.

## II. MATERIALS AND METHODS

**Figure 2:**
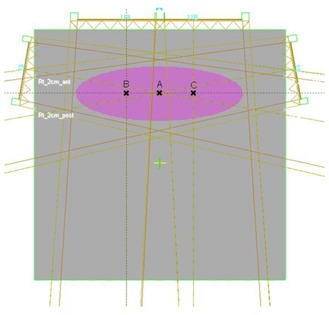
Schematic diagram of the dose optimization and calculation geometry used in this work. The central transverse slice of the geometry is shown. The phantom is cubic $\left(30\times 30\times 30\;{\text{cm}}^{3}\right)$ with a cylinder of elliptical cross section as the target. The major and minor axes of the ellipse are 20 cm and 6.6 cm, respectively, and its length is 7.8 cm, resulting in a volume of 793.3 cm^3^. Point A is centered in the target volume and is the isocenter for all lateral oblique fields, and for anterior fields delivered with multiple carriage positions. Points B and C, 8 cm apart, are isocenters for cases where the anterior component of the dose distribution is delivered with dual isocenter fields.

A commercial treatment‐planning system (Eclipse, version 6.5, build 7.1.59, Varian Associates, Palo Alto, CA) was used for optimization and isodose distribution calculation for all IMRT treatments in this study. Figure 2 shows the central transverse slice of the dose optimization and calculation geometry. The phantom is cubic $\left(30\times 30\times 30\;{\text{cm}}^{3}\right)$, embedded with a cylinder of elliptical cross section as the target. The major and minor axes of the ellipse are 20 cm and 6.6 cm, respectively, and its length is 7.8 cm, resulting in a volume of 793.3 cm^3^. Point A is centered in the target volume and is the isocenter for all lateral oblique fields, and for anterior fields delivered with multiple carriage positions. Points B and C, 8 cm apart, are isocenters for cases where the anterior component of the dose distribution is delivered with dual isocenter fields. A three‐field beam arrangement was selected (one anterior and two lateral‐oblique beams with an angle of 160° between them) to treat this volume. All beams had a nominal energy of 6 MV. Dose constraints were chosen to give 100% dose throughout the planning target volume (PTV), with a hotspot of 110%. After optimization, the plans were considered acceptable if 95% of the PTV volume received 95% of the dose. The width of the target was intentionally made large so that treatment was not possible with a single carriage position for the anterior field. The rationale of having a longer width was to ensure that the treatment‐planning system would break up the optimal fluence to be delivered with multiple carriage positions. Thus, even though the PTV volume is not very large, the results are still meaningful. A very large volume would have just increased the computation time for optimization and forced us to use a larger calculation grid. The results from the study of this smaller anatomy, in our opinion, pertain to the treatment of larger volumes as well.

Multiple treatment plans were generated and dose statistics were compared. Plans were designed with a single anterior field with dual carriage positions, or with the anterior field split into two fields with separate isocenters 8 cm apart with the beams being forcibly matched at the isocenter or with 1 cm, 2 cm, 3 cm, and 4 cm overlap. Deliveries in both step and shoot (SMLC) and sliding window (DMLC) modes were studied. In all treatment plans, jaw positions were manually fixed before starting optimization, and dose constraints remained unaltered. Optimizations were allowed to continue for an equivalent number of iterations or until no change in the optimization function was noticed. Deliverable photon fluences were then determined using a leaf‐motion calculator, and full volumetric dose computations were subsequently carried out with a calculation grid size of 0.25 cm.

## III. RESULTS AND DISCUSSION

**Figure 3:**
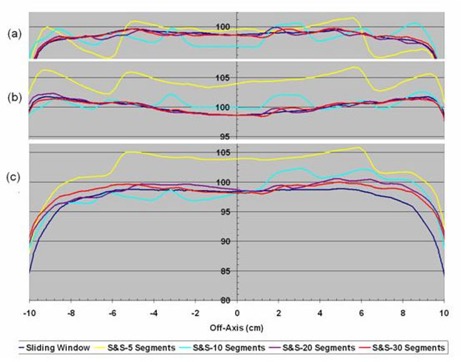
Off‐axis dose profiles in planes (a) 2 cm anterior to isocenter, (b) containing the isocenter, and (c) 2 cm posterior to isocenter taken from dose distributions calculated for sliding window or step and shoot IMRT with 5, 10, 20, and 30 segments, all delivered with dual carriage positions.

**Figure 4:**
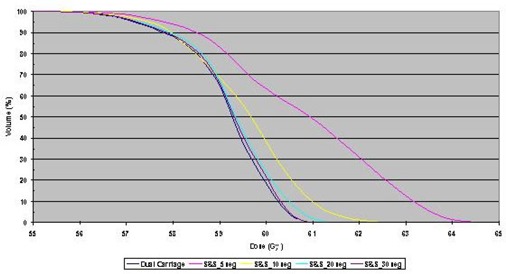
Dose‐volume histograms (DVHs) for dose distributions calculated for sliding window or step and shoot IMRT with 5, 10, 20, and 30 segments, all delivered with dual carriage positions. In all DVHs, 100% corresponds to 60 Gy.

The homogeneity in the overlap region of a fluence delivered with multiple carriage positions was studied. Profiles through isocenter and 2 cm anterior and posterior to the isocenter plane for DMLC and SMLC treatment plans with dual carriage positions are shown in Fig. 3. The step and shoot data are for 5, 10, 20, and 30 segments per carriage position, while the sliding window treatment plan uses 320 control points for each carriage position. The corresponding DVHs are shown in Fig. 4. It is clear that the DMLC plan is the most uniform and best satisfies the dose constraint; however, similar dose uniformity can be achieved with SMLC delivery provided that at least 20 segments per carriage position are used. With fewer segments, the dose statistics become suboptimal.

**Figure 5:**
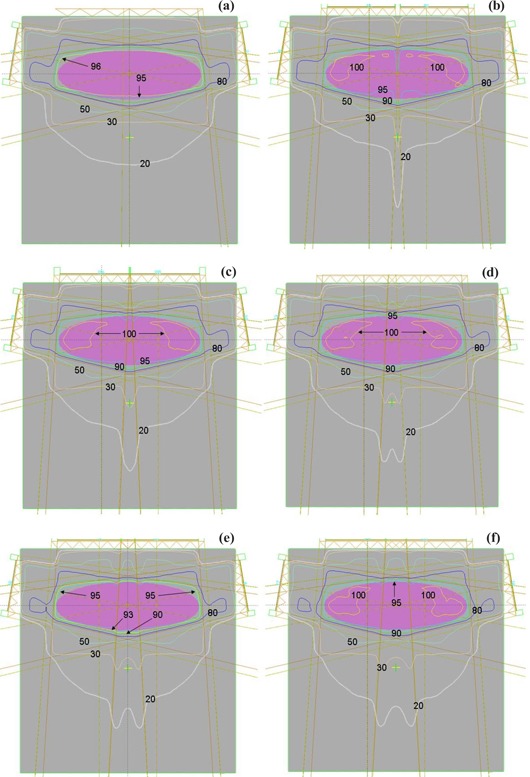
Isodose distribution calculated for delivery with (a) dual carriage position sliding window, (b) dual isocenter for the anterior field with no field overlap, and (c) to (f) dual isocenter for the anterior field with 1 cm, 2 cm, 3 cm, and 4 cm overlap, respectively.

It has been shown that splitting optimal fluences for delivery with multiple carriage positions works well for both DMLC and SMLC delivery. In the case of very wide fields that cannot be treated with multiple carriage positions, multiple isocenters can be used to cover the target volume. It is instructive to compare dose statistics for multiple isocenter plans with those achieved with multiple carriage positions. Multiple treatment plans for the PTV described above were evaluated. As stated previously, the target volume required dual carriage positions for the anterior field, and similar plans were calculated with dual isocenters with varying overlap of the fields within the target volume. Figure 5(a) shows the isodose distribution calculated for a dual carriage DMLC delivery that satisfies all dose constraints. To study dose delivery with dual isocenters, see Figs. 5(b), (c), (d), (e), and (f), which show isodose distributions through a central transverse cut of the phantom with 0 cm, 1 cm, 2 cm, 3 cm, and 4 cm overlap, respectively, between fields. From the figures, it is clear that all the dual isocenter plans had slight underdosage anterior to the plane containing the isocenters, along with slight overdosage posterior to this plane. The dual carriage plan, however, produced a more uniform dose throughout the target volume without the accompanying hot or cold spots.

**Figure 6:**
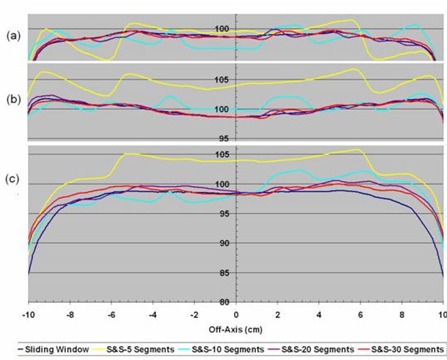
Off‐axis dose profiles in planes (a) 2 cm anterior to isocenter, (b) containing the isocenter, and (c) 2 cm posterior to isocenter taken from dose distributions calculated for delivery of the anterior field with dual carriage position sliding window or dual isocenter with 0 cm, 1 cm, 2 cm, 3 cm, and 4 cm field overlap.

To compare dual isocenter and dual carriage position delivery further, it is instructive to examine dose profiles throughout the target volume. Figure 6 shows the off‐axis profiles for the dual carriage case within the isocenter plane and both 2 cm anterior and 2 cm posterior to it. Also shown in Fig. 6 are the respective off‐axis profiles of DMLC plans with dual isocenters and 0 cm, 1 cm, 2 cm, 3 cm, and 4 cm overlap. The dual carriage plan demonstrates the best uniformity, while the dual isocenter profiles show the expected hot and cold regions. For the dual isocenter plans, abutting fields result in the greatest nonuniformity, while an overlap of 4 cm produces a profile closest to the dual carriage plan.

**Figure 7:**
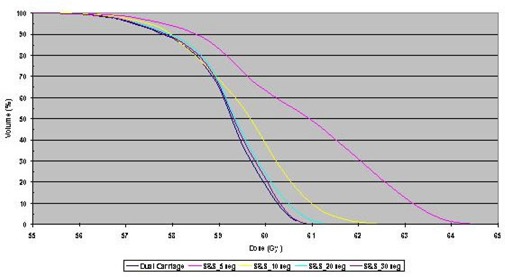
Dose‐volume histograms (DVHs) for dose distributions calculated for delivery of the anterior field with dual carriage position sliding window or dual isocenter with 0 cm, 1 cm, 2 cm, 3 cm, and 4 cm field overlap. In all DVHs, 100% corresponds to 60 Gy.

Figure 7 shows DVHs for dual carriage as well as dual isocenter IMRT beams with no overlap, 1 cm, 2 cm, 3 cm, and 4 cm overlap, respectively. As can be seen from Fig. 7, there were significant differences in the DVHs for all the plans, with the dual carriage plan best satisfying the constraints. Among the dual isocenter plans, the one with 4 cm overlap was found to be closest to the dual carriage plan. Since multi‐isocenter plans are necessary for large‐volume IMRT treatments due to the MLC design constraints discussed above, it is imperative to select an appropriate overlap between fields in a dual isocenter IMRT plan. As can be seen from Figs. 5 to 7, the dual isocenter plans with the greatest overlap region produced the plan most similar to the dual carriage plan. However, an overlap takes away from the field width that can be treated, so a compromise must be made between a large overlap to produce the best uniformity and a small overlap to give the widest possible coverage. Study of the off‐axis profiles and the DVHs for all the above cases reveals only a marginal improvement in going from a 3‐cm overlap to a 4‐cm overlap. Thus, in those cases where dual isocenters are required, an overlap of at least 3 cm between fields should be used. Furthermore, many large IMRT fields are used in the abdominal region, where there can be a 1‐cm to 2‐cm difference in the anterior‐posterior separation during normal breathing. For ungated treatments, a minimal overlap could result in significant cold spots within the target volume. Hong et al.^(5)^ have similarly suggested an overlap of 3 cm when treating the whole abdomen with IMRT.

As with field matching in conventional radiotherapy, dose nonuniformities within the match region for dual isocenter IMRT were greatest when fields were abutted. As the overlap was increased, field uniformity improved. In reality, increasing the overlap region essentially shifts the point of abutment anterior to the original isocenter. Thus, it is possible to move the field abutment outside the PTV by selecting an appropriate overlap, consequently increasing dose uniformity within the PTV. While this may slightly increase the hotspot within the PTV (usually clinically acceptable), the magnitude of the overdosage beyond the PTV is decreased, potentially sparing dose‐sensitive organs outside the PTV.

One alternative to multiple isocenters for treating large target volumes is to use extended SSDs. This technique, however, has several significant drawbacks. First, IMRT, particularly with the sliding window approach, is already relatively inefficient from a dose delivery point of view. Increasing the treatment distance will further lower the gray‐to‐monitor unit ratio and result in longer treatment times with greater potential for patient movement. Second, extended treatment distances bring inefficiency in patient and field setup, since the treatment fields are no longer delivered isocentrically, thereby prolonging the overall appointment time and affecting patient throughput. Third, at extended distances, the MLC leaves are magnified, degrading the conformity of the MLC‐defined field around not only the PTV but also any critical structures. Fourth, the SSD nature of the extended distance amplifies setup errors, which can affect the quality of treatment. Fifth and finally, it has been suggested that when the time necessary to deliver a fraction of radiation therapy exceeds 30 min, the radiobiological effectiveness of the dose can be compromised.^(6)^ Therefore, the increase in treatment time for an extended SSD IMRT dose delivery may result in suboptimal tumor control. Taking all the above issues into consideration, the multiple isocenter approach to treatment of large target volumes with IMRT may be the best solution.

## IV. CONCLUSION

Large target volumes can be effectively treated with IMRT using either multiple carriage treatment delivery or multiple isocenters. Furthermore, if the particular algorithm used for IMRT planning does not have the capability of splitting optimal fluences for delivery by multiple carriage positions, or the MLC itself cannot deliver split fluences, it may be necessary to use multiple isocenters for even smaller target volumes. When contemplating multiple carriage delivery in both step and shoot and sliding window modes, the number of subfields selected will have an impact on the uniformity of the dose within the target volume. For target volumes that are too large to be treated with multiple carriage positions, dual isocenters should be selected over extended treatment distance. It has been demonstrated that dual isocenter delivery can result in clinically acceptable plans provided some thought is put into the amount of overlap between adjacent fields.
